# The Health and Lives of Dental Professionals in the Brazilian Public Health System During the COVID‐19 Pandemic: An Observational Study in the City of Manaus, Amazonas

**DOI:** 10.1155/ijod/1549178

**Published:** 2025-12-28

**Authors:** Priscilla Farias Naiff, Shirley Maria de Araújo Passos, Tania França

**Affiliations:** ^1^ School of Public Health, Municipal Health Secretariat of Manaus, Manaus, Amazonas, Brazil; ^2^ School of Health Sciences, Amazonas State University, Manaus, Amazonas, Brazil, uea.edu.br; ^3^ Institute of Social Medicine, Rio de Janeiro State University, Rio de Janeiro, Brazil, uerj.br

**Keywords:** COVID-19, dentistry, public health

## Abstract

**Objective:**

To investigate the implications of the COVID‐19 pandemic on the lives of dentists in the municipal health network of Manaus.

**Methods:**

A cross‐sectional, descriptive, and exploratory study with a quantitative and qualitative approach. Between April and July 2022, an online questionnaire with closed and open questions was applied to dentists. The study included 81 professionals. The collected data were stored in a Microsoft Excel 2007 spreadsheet, generated by Google forms. For categorical data, absolute and relative frequencies were obtained. Content analysis, aided by Atlas ti software, Version 22, was used for textual analysis.

**Results:**

The COVID‐19 pandemic represented a constant source of stress and fear for dentists, negatively affecting their mental health.

**Conclusions:**

In view of the above, the Unified Health System (SUS) needs to implement effective measures to protect and promote the mental health of its workers, such as offering specialized psychological support, promoting awareness campaigns on mental health risks, and creating support groups for professionals.

## 1. Introduction

COVID‐19 is a disease caused by severe acute respiratory syndrome coronavirus 2 (SARS‐CoV‐2), which belongs to the Coronaviridae family and the Nidovirales order. First identified in Wuhan, China, in late 2019, the virus became popularly known as the “novel coronavirus” [1].

After the confirmation of the first case of COVID‐19 in Brazil, originating in the State of São Paulo, the disease quickly spread throughout the country, devastatingly affecting the city of Manaus, Brazil, making it one of the capitals most affected by the pandemic. The city experienced one of the most alarming scenarios related to COVID‐19, exposing Brazil and the world to the collapse of public health services, with the overwhelmed care network and the shortage of oxygen for patients admitted to hospitals, resulting in a high number of deaths. According to data from the Amazonas Health Surveillance Foundation [2], during the first and second peaks of the disease in 2020 and 2021, the occupancy rate of Intensive Care Unit (ICU) beds reached 96% and 90%, respectively. In November 2021, this rate was around 32%.

SARS‐CoV‐2 is generally transmitted through the inhalation of respiratory droplets or aerosols expelled by infected individuals, mainly during sneezing, coughing, or talking. Direct contact with the oral, nasal, or ocular mucosa is also a route of transmission. It is worth noting that these aerosols can remain suspended in the air for a certain amount of time or settle on nearby surfaces, posing a risk of contagion. Therefore, physical contact with contaminated objects or people can also lead to infection [3].

The high degree of dissemination of SARS‐CoV‐2 and the vast volume of information released in the context of the pandemic, often a lacking scientific basis, have caused changes in the population’s behavior, psychological suffering, uncertainty, insecurity, and mental disorders [4].

Dentists, due to the nature of their work, are in direct contact with the oral cavity of patients, exposing themselves to saliva, blood, and aerosols generated during most clinical procedures. This close, almost face‐to‐face interaction exposes these professionals to an extremely high risk of contracting the coronavirus [5], and this high risk can significantly affect the lives of these workers, especially in relation to mental and general health.

During the COVID‐19 pandemic, dentists within Brazil’s public health system faced unique challenges that warrant an in‐depth study of its impact. The nature of public care, which is characterized by high demand and often limited infrastructure, was drastically altered by the need for rigorous biosafety measures. The scenario in Manaus, in particular, with its healthcare system on the brink of collapse, intensified these difficulties. Municipal Unified Health System (SUS) dentists in Manaus faced the suspension of elective procedures, the reallocation of services to treat exclusively urgent and emergency cases, and the need to adapt their practices with scarce resources. Furthermore, many were integrated into COVID‐19 response efforts that extended beyond routine dental care, such as the collection of biological samples for testing. This abrupt shift in work processes, coupled with the high risk of contamination, exposed these professionals to a distinct form of operational and psychological stress. Therefore, analyzing their experiences is fundamental to understanding the repercussions of the pandemic on public oral healthcare services.

Considering the context of the COVID‐19 pandemic and its impacts on dentistry, this study aimed to identify the repercussions of the pandemic on the lives of dentists in the municipal health network of Manaus.

## 2. Material and Methods

This cross‐sectional, descriptive, and exploratory study, with a quantitative and qualitative design, was conducted in Manaus, the capital city of the state of Amazonas. This study covered four Health Districts (DISAs) of Manaus: North, South, East, and West. A total of 116 health establishments that offered dental care to the population were included. DISA Rural was excluded due to the peculiar characteristics of dental work in this area, with teams working in both terrestrial and riverside areas.

### 2.1. Application of the Questionnaire

An exploratory field survey was conducted in Manaus, aiming to map the particularities, practices, challenges, and suggestions of dentists working in SUS dental services. To this end, a semi‐structured questionnaire was applied using the Google Forms platform. The questionnaire, sent via email and messaging application, was prepared based on previous studies [6–8]. Table 1 presents a summary of the survey’s thematic blocks. The study included dentists who worked in municipal public health care, linked to Basic Health Units (BHUs), Family Health Units (FHUs), polyclinics, or Dental Specialty Centers (DSCs), in the urban area of the municipality of Manaus between April and July 2022. The selection of participants occurred through free and spontaneous agreement by signing a Free and Informed Consent Form (FICF), made available via Google Forms. It is important to mention that this study excluded professionals who performed administrative or managerial activities.

**Table 1:** Identification of survey blocks.

Category	Subcategory
Block 1—Profile of dentists	Gender, age, education, time since education, time of service in the SUS, specialty at work.

Block 2—Implications of the pandemic on one’s personal life	Immunization status, manifestation of COVID‐19, repercussions of the pandemic on the personal lives of the interviewees.

Block 3—Opinions	Repercussions of the pandemic on the lives of professionals.

*Note: Source*: Created by the author (2024).

The study population consisted of 287 professionals as of May 2022, according to data from the National Registry of Health Establishments (*Cadastro Nacional de Estabelecimentos de Saúde—*CNES) of the Ministry of Health [9]. Adopting a 95% confidence interval (95% CI) and a 5% sampling error, a representative sample of 165 participants was obtained from the sample calculation [10].

To reach the research participants, contacts were established with the oral health management and technical support departments of the health districts. These, in turn, placed the researcher in contact with the dentists.

### 2.2. Analysis of Results

In total, 177 dentists were contacted. Of these, only 82 (46.3%) responded to the questionnaire. One professional was excluded from the study because he worked in the managerial area. It should be noted that no participant failed to answer any of the questions. The professionals contacted who did not respond to the survey were considered as having refused to participate.

The data from the closed questions of the online questionnaire were stored in a Microsoft Excel 2007 spreadsheet generated by Google Forms. Statistical analyses were conducted using GraphPad Prism, version 10.6.0 (GraphPad Software, San Diego, California, USA). The absolute number and frequency were calculated for the categorical data. To analyze the associations between categorical variables and cross‐tabulation, and for impacts with sufficient sample counts, the chi‐square test of independence was used, while Fisher’s exact test was employed for those with low frequencies. To quantify the magnitude of the association, effect sizes were calculated using the Phi (Φ) and Cramer’s V (V) coefficients, and the 95% CI was reported for each estimate. Additionally, the Two One‐Sided Tests (TOST) for equivalence was applied to confirm if the absence of significant differences represented functional equivalence between groups, with the practical relevance margin set at an effect size less than ∣Φ∣ < 0.20 or ∣V∣ < 0.20. The statistical significance level was set at *p*  < 0.05.

The open questions of the questionnaire were subjected to content analysis in order to generate inferences, using the Atlas.TI software, version 22. To this end, the following steps were employed [11, 12]:

1. Pre‐exploration of the material: The responses to the interviews were read carefully, enabling the organization of the content relevant to the study.
2. Selection of the units of analysis: Excerpts from the interviews whose meanings, taken together, could provide context for the researcher’s inferences were highlighted and categorized. After categorizing the responses, the absolute and relative frequencies of these results were obtained and presented in the tables.
3. Categorization of responses: The responses were categorized into
  a. Negative repercussions of the COVID‐19 pandemic on the personal lives of dentists: addressed the negative impacts of the pandemic on the personal lives of professionals;
  b. Positive repercussions of the COVID‐19 pandemic on the personal lives of dentists: focused on the positive aspects of the pandemic on the personal lives of professionals;
  c. No repercussions on personal lives: included responses that did not indicate impacts of the pandemic on the personal lives of the professionals.
4. Data analysis and interpretation: The absolute and relative frequencies of the categories were obtained and presented in Table 2. Next, the results were interpreted and discussed in light of the theoretical framework used for this study.
5. Data saturation: This is a concept of methodological rigor that guides qualitative data collection. Its use is justified by ensuring that the sample size is sufficient to explore the phenomenon in depth without the need for excessive data collection. Guest et al. conducted a thematic analysis of 60 interviews with health professionals. The striking result was that with just 12 interviews, the research team had already captured most of the common and relevant themes. By the 20th interview, virtually all essential themes had emerged. Thus, this study was based on Guest et al.’s study [12], considering thematic saturation to be reached with 12–20 interviews.

**Table 2:** Specialty of dentists in the municipal health network, Manaus, Amazonas, 2022.

Specialty	*n*	%^a^
General practice (no specialty)	62	76.4
Oral and maxillofacial surgery	7	8.6
Endodontics	9	11.1
Pediatricdentistry	6	7.4
Orthodontics	4	4.9
Patients with disabilities	9	11.1
Periodontics	6	7.4
Prosthetics	7	8.6

*Note: Source:* Prepared by the author based on the responses to the questionnaire (2024).

^a^More than one answer option. The sum of the percentages does not necessarily have to be 100%.

### 2.3. Ethical Precepts

This study began after approval by the Research Ethics Committee of the State University of Amazonas (CAAE 55987822.3.0000.5016), in accordance with Resolution No. 466/2012 of the National Health Council (NHC). This resolution approves the Guidelines and Regulatory Standards for Research Involving Human Subjects. Furthermore, the research followed NHC Resolution No. 510/2016, which establishes the standards applicable to research in the humanities and social sciences that use data collected directly from participants. Finally, the study also followed the guidelines for research in a virtual environment contained in Circular Letter No. 1/2021‐Conep/SECNS/MS.

## 3. Results and Discussion

The questions related to the interviewed dentists regarding their reports of contamination and vaccination against the novel coronavirus were analyzed. It should be noted that this study did not confirm the diagnosis of COVID‐19 through laboratory tests or by requesting proof of vaccination from the participants. In addition, it was verified how the pandemic impacted the personal lives of the interviewees, observing whether it generated changes in behavior and routine, as well as what the negative and positive implications were for these people.

### 3.1. Profile of Dentists Working in Dental Care Within the Municipal Health Network

Initially, data regarding gender, age, degree, and time since graduation, as well as time and area of practice in SUS, were analyzed with the aim of outlining the profile of the dentists who participated in this study.

A total of 81 participants who agreed to participate in the study were included, with an average age of 45.3 years, 72.8% of whom were female and 27.2% male.

Regarding the sample size, several characteristics of the respondents may have significantly influenced the response rate, resulting in the failure to reach the calculated number (*n* = 165), such as a lack of interest or knowledge about the subject addressed in the questionnaire, a lack of time, emotional availability to participate, or resistance to participating in online surveys. It is likely that the excessive number of surveys previously received by professionals, as well as the sending of very long questionnaires, created barriers to participation in future studies [13].

It is important to note that a sample of 81 participants exceeds typical saturation thresholds for qualitative research [12], and the percentage of responses achieved was capable of providing important data for describing the regional panorama, taking into account the necessary precautions for generalizing the data.

The sociodemographic profile of the participants showed a predominance of female dentists, with an average age of 45 years. This profile is in line with another study conducted with dentists at the local level [14].

It is important to note that none of the participants had less than 6 years of training or experience as a dentist in SUS. The study participants were mostly experienced professionals, with more than 11 years of training and experience in SUS, and about half had been dentists for more than 20 years (Table 3).

**Table 3:** Distribution of dentists, according to sex, time since training, and experience in SUS, Manaus, Amazonas, 2022.

Time (years)	*n* of dentists (%) education	*n* of dentists (%) experience
6–10	3 (3.7)	26 (32.0)
11–15	17 (21.0)	17 (21.0)
16–20	21 (25.9)	19 (23.5)
>20	40 (49.4)	19 (23.5)
Total	81 (100)	81 (100)

*Note: Source:* Prepared by the author based on the responses to the questionnaire (2024).

Regarding education, the majority of participants (96.3%) had a postgraduate degree, with at least a specialization, followed by the same percentage (17.3%) of master’s and Ph.D. recipients. Only 3.7% of these professionals had no postgraduate education at the time of the study (Table 4).

**Table 4:** Maximum level of training of dentists working at SEMSA, Manaus, Amazonas, 2022.

Type	*n*	%
Undergraduate	3	3.7
Specialization	50	61.7
Master’s	14	17.3
Doctorate	14	17.3
Total	81	100

*Note: Source:* Prepared by the author based on the responses to the questionnaire (2024).

This study obtained results that were similar to findings from Passos [14], who identified that more than half of the dentists who worked in Primary Health Care (PHC) in the metropolitan region of Manaus held the title of specialist. Among the areas of specialization cited by Passos, 37% of the participants had a specialization in collective health or public health. Thus, the author highlighted the importance of this training for professionals who work in the family health strategy, as these specialties provide support for healthcare work provided to families, combined with management, planning, and evaluation actions, with greater expertise in the services offered to the community [14]. Table 2 shows that most of the interviewees worked in the general clinical area, which is the responsibility of the PHC in the municipal network, with activities related to the general clinic, such as prevention and promotion of oral health, educational activities, dental emergencies, restorative dentistry, and minimally complex tooth extractions. Among these professionals are those who worked in parallel in specialized care, depending on the number of private positions the health professionals held. Others worked exclusively with the DSCs, which offer the population specialized care in oral surgery, endodontics, pediatric dentistry, orthodontics, patients with disabilities, periodontics, and dental prosthetics.

### 3.2. Manifestation of COVID‐19 by Dentists

A positive diagnosis for SARS‐CoV‐2 was reported by 79% (*n* = 64) of the respondents and a negative diagnosis by 21% (17) of the participants between March 2020 and July 2022. Among the positive diagnoses up to the time of the interview, 76% (49), 23% (15), and 1% (1) had contracted the disease once, twice, and three times, respectively. Regarding the period of the pandemic in which this diagnosis was made, 43.8% (28) reported COVID‐19 in 2020, 45.3% (29) in 2021, and 37.5% (24) by July 2022.

Among the participants who had COVID‐19, 89.1% (57) reported having maintained only home isolation, 3.1% (2) required hospitalization, and 7.8% (5) did not maintain any type of isolation during the period of the disease.

These data possibly indicate that the symptoms related to the disease were mild in this population. However, it is worth mentioning that this study did not survey the number of dentists in the municipal health network who were hospitalized and died (which is part of the frequency of dentists who were hospitalized).

The frequency of the disease among dental professionals in this study was generally much higher than that evidenced by other studies involving health professionals.

In a study carried out in Paraná [15] with health professionals who worked at a referral hospital that treated patients with COVID‐19, the detection rate for SARS‐CoV‐2 was 12.8%, while a study carried out in Spain observed that 13% of the cases that tested positive for the virus were among health professionals [16].

A systematic review and meta‐analysis of 94 studies carried out with 127,480 health professionals from North America, Europe, Africa, and Asia, between March and June 2020, showed an overall seroprevalence of 8.7% of SARS‐CoV‐2 antibodies. All but one of the studies used convenience samples, and most were conducted in hospitals or primary care centers [1].

One study conducted in the United States indicated a prevalence of 4.4% of SARS‐CoV‐2 among 24,000 healthcare workers between April and August 2020. Community contact with COVID‐19 was associated with seropositivity, but not with one’s role in the workplace, environment, or contact with COVID‐19 patients. Prolonged contact with patients and aerosol production, however, were not assessed in this study [17].

In another study also conducted in Brazil, there was a similar prevalence of infection among dentists (19.1%) and in the population of the Federal District of Brasília (17%). However, this finding applies to the epidemiological situation in 2020, before the development of vaccines and the emergence of the SARS‐CoV‐2 Delta variant [18].

In a university hospital in Rio de Janeiro during the COVID‐19 pandemic in 2020, the prevalence of SARS‐CoV‐2 infection among health professionals was 44.2% [19]. These data corroborate the prevalence found in this study (79%). However, in the present study, the seroprevalence of antibodies against coronavirus was not evaluated, only the self‐reported diagnosis of COVID‐19 by the participants. In addition, due to the lower participation of dentists in the study than expected, if the estimated sample size had been reached, the difference in the number of participants could influence the results. It is also important to note that detecting the prevalence of coronavirus infection by dentists in the municipal network of Manaus was not within the scope of the present study.

One of the possible explanations for the high frequency of COVID‐19 in this study may be occupational transmission, especially at the beginning of the pandemic, given the redirection of dentists to care for patients with COVID‐19, as well as the lack or possible incorrect use of personal protective equipment (PPE), which may have increased the chain of viral transmission [1].

Another plausible possibility associated with this high frequency of coronavirus contamination reported by these professionals may, in fact, be more related to the general scenario of the COVID‐19 pandemic (community transmission) in Manaus rather than to occupational factors. The state of Amazonas was the center of attention on social media during the critical periods of the pandemic, as Manaus was one of the cities that suffered the most from its impacts at a national level. After the end of the first wave of the disease, in mid‐2020, many people began to have a false sense of protection, influenced by the discourse of “herd immunity” [20, 21], which led to a relaxation of measures to combat the virus, such as reduced social distancing and hygiene measures, including hand washing and the use of masks by the general population.

At the end of 2020, with the number of cases increasing sharply, along with a significant increase in the percentage of hospitalizations in the latter half of December, it was considered that some other factor may well exist, different from that of the first wave of the disease and the reduction of immunity against SARS‐CoV‐2 infection in previously exposed individuals, which could be involved in the significant increase in new cases [20]. It is possible that the P1 lineage variant that emerged at that time, with greater transmissibility than preexisting lineages, was a cofactor responsible for the explosion of cases in the city [20, 21].

In view of the above, it can be assumed that if early restrictive measures with intersectoral action planning, including health, economy, and social assistance, population awareness, and greater adherence to the measures imposed by the State, had been implemented by the government in Manaus, it is likely that the second wave of the disease would have been better addressed [22, 23] without the catastrophic effects resulting from failures observed during the process of preventing contagion by the coronavirus and in combating the disease, regardless of the emergence of a new variant.

Non‐pharmacological measures are essential in order to combat the virus. The non‐adherence of the Amazonian population is related to such factors as vulnerable populations. In Manaus, great extremes are observed, with the concentration of wealth in the hands of a few and a large portion of the population living in poverty. These people could not stop working because there was no other way to provide the basic needs for their households. Furthermore, the presence of groups in various spheres of government and society that questioned scientific knowledge generated a feeling of uncertainty among the population [24–26].

In a study conducted in the Federal District of Brasília with dentists from the private or public sector who continued to provide dental care with reduced working hours, seropositivity for SARS‐CoV‐2 in these professionals was associated with a confirmed diagnosis of COVID‐19 in a family member, suggesting a possible SARS‐CoV‐2 infection due to contact with a family member or community transmission, rather than nosocomial transmission in a dental office [18].

In Manaus, elective dental care was suspended before the first peak of the pandemic, between April and May 2020, and only resumed (partially) in March 2021, after the second peak. Even in the permitted emergency care, the use of aerosols was avoided whenever possible. Perhaps, if there had not been a suspension and/or restrictions on dental care, the percentage of contamination (and deaths) by SARS‐CoV‐2 would have been higher and more catastrophic among oral health professionals, especially at the beginning of the pandemic, before the development of vaccines against the virus and its variants.

The prevalence of SARS‐CoV‐2 among health professionals is high. Excellent adherence to infection prevention and control measures is necessary, such as the use of adequate and sufficient PPE. Early recognition, identification, and isolation of healthcare professionals infected with SARS‐CoV‐2 are imperative in order to reduce the risk of infection by the virus in this professional environment [1]. In a study where the rate of SARS‐CoV‐2 infection observed among dentists was similar to that of the general population, the authors suggest that the use of PPE during patient care may be responsible for preventing dentist contamination. Disease prevention was associated with the new routine of dental practice, such as reinforcing biosafety measures, improving physical barriers, reducing aerosol production, monitoring signs and symptoms of patients associated with COVID‐19, and the frequent testing of patients and the dental team [18].

One fact that draws attention in the study is that some dentists (7.8%) reported being infected with the coronavirus at some point during the pandemic without having undergone self‐isolation at home or in a hospital. This is extremely worrisome, as these professionals may have acted as agents of spreading the virus in the workplace and in society.

In view of the above, it is extremely important to emphasize the need for a broad dissemination of scientific knowledge about COVID‐19 and other topics, not only in scientific journals, but also on social media and other means of direct communication with society, such as radio, television, newspapers, and magazines. The fight against fake news, or false information disseminated as news on social media and generally sensationalist, must be carried out more openly so that it ceases to confuse the general population. Health professionals must always gear their clinical practices and conduct towards society and base their work environment on the evidence available in the scientific literature. Fake news impacts people’s lives and health, how individuals understand the health/disease process, and how they deal with issues related to prevention. The risk of harm from fake news is difficult to estimate, given its potential for dissemination, combined with a cultural and political context marked by an ideological war that has divided society, polarizing it and causing fear and distrust in political and media institutions—a condition that catalyzes hate speech, conspiracy theories, and smear campaigns. It is a problem that requires adequate confrontation through public policies [27].

### 3.3. Immunization Status of Dentists

Manaus was one of the pioneering cities in Brazil to implement vaccination more extensively [28], starting on January 18, 2021. Vaccinometer data show that, in relation to the vaccinable population, coverage of ~77% was achieved relative to the two‐dose cycle of the vaccine, the minimum recommended by the MS [29].

Among all 5085 registered dentists in Manaus, as of December 31, 2022, 70% had completed two doses, according to data from the Regional Dental Council (RDC) [29]. In contrast, our 81 SUS‐network respondents reported that as of July 2022, 92.6% (75) of the participants reported having at least the three‐dose vaccine schedule and 100% two‐dose coverage. The high vaccination coverage observed in our sample contrasts sharply with that of the general dentist population in Manaus.

This discrepancy is largely explained by Manaus’s Municipal Decree 5.146 of 2021, which made vaccination mandatory, with at least two doses of the vaccine, for all municipal public agents, which indicates that all professionals followed the governmental determination. Consequently, the sample is highly homogeneous and is only representative of dentists working in the municipal network, not reflecting the entire professional class in the city. The lower coverage among the general dentist population may be attributed to factors such as controversial political opinions regarding herd immunity and early treatment, as well as the logistical and structural difficulties faced by Manaus, which was a pandemic epicenter [30]. This difference suggests a selection bias, thereby limiting the generalizability of our findings to professionals not working in public service.

Throughout history, no disease has ever been contained through collective or herd immunity. Smallpox and measles, for example, were only controlled after the advent of vaccines. Talking about early treatment or collective immunity in the management of COVID‐19 makes it difficult to control the disease, diminishing society’s general trust in social distancing measures [30].

It was observed that, after vaccination, even with the resumption of elective care in April 2021, there was no significant increase in the number of COVID‐19 cases in the studied population (43.8%, *n* = 28, in 2020 and 45.3%, *n* = 29, in 2021) and, in fact, there was a reduction in 2022 (37.5%, *n* = 24), although insignificant (*p*  > 0.05).

Immunization schemes using the Coronavac (96.3%, *n* = 78), Pfizer (87.7%, *n* = 71), and Astrazeneca (4.9%, *n* = 4) vaccines were reported in the study participants. However, it is not possible to answer whether or not there was an association of these vaccines related to the three doses, as this was not within the scope of this study.

### 3.4. Use of Free or Leisure Time During the Pandemic

The results showed that most professionals dedicated their free time to activities related to family life (66.7%; *n* = 54), watching television (45.7%; 37), or hobbies such as guitar, art, sewing, music, and cooking (19.8%; 16). There was also a report of dedicating time to physical activities (19.8%; 16) as well as reading (35.8%; 29) or online courses and conferences in dentistry (43.2%; 35). These data corroborate a study carried out with medical students during the critical period of the pandemic, in which it was reported that many of the students focused on professional training, greater experience with and development of family ties, leisure activities, and individual reflections on many aspects of daily life [31].

### 3.5. Implications of the Pandemic on the Personal Lives of Dentists

The professionals reported that the pandemic had numerous repercussions on their lives, with implications for personal aspects of their behavior and routines. In general, these repercussions were related to negative or positive aspects in the opinion of the interviewees, according to Table 5:

**Table 5:** Repercussions of the COVID‐19 pandemic on the personal lives of dentists, Manaus, Amazonas, 2022.

Aspects	*n*	%^a^
Negatives		
Damage to mental health	29	35.8
General health complications	8	9.8
Social isolation	9	11.1
Loss of family members	8	9.8
Loss of purchasing power	2	2.4
Overload of obligations	2	2.4
Positives		
Sharpened sense of community	1	1.2
Acquisition of new knowledge	2	2.4
Acquisition or improvement of good habits	14	17.2
No impact on personal life	4	4.9

*Note: Source:* Prepared by the author based on the responses to the questionnaire (2024).

^a^More than one answer option. The sum of the percentages does not necessarily have to be 100%.

Among the negative aspects associated with changes in the private lives of professionals, the following can be mentioned: mental health shock, such as living in a constant state of alert, anguish, worry, depression, anxiety, insecurity, fear, panic, stress, family crises, trauma, or feeling of nonconformity; general health complications, such as a sedentary lifestyle, hospitalizations due to COVID‐19, memory loss, pain in different parts of the body, abnormalities with blood pressure, shortness of breath, weight gain; social isolation—avoiding crowds, social distancing, drastic reduction in contact with family members, fear of traveling and going to places with a large crowds; the loss of family members or friends due to lack of beds or complications from COVID‐19; a loss of purchasing power due to a reduction in family income, as some dentists worked in the private sector to supplement their income and had to stop their activities; an increase in inflation; an overload of obligations, such as those related to work and household chores; and the impossibility of resting on vacation, as they were suspended by the public administration.

There were changes in behavior and routine for dentists, such as physical and mental health problems, social isolation, the loss of family and friends, a loss of purchasing power, and an overload of obligations. The impact on mental health was also reported in another study that evaluated psychosocial factors of Brazilian dentists, faced with the risk of SARS‐CoV‐2 infection, in the public and private sectors. It was reported that the pandemic was a time of tension and many concerns for families. The professionals participated in the screening of COVID‐19 patients in which the risk of coronavirus contamination was high. The participants’ mental health was significantly affected, and some professionals reported having symptoms of anxiety, insomnia, deep sadness, discouragement, fear, anguish, and pain [32]. Other studies show that significant psychological stress related to the risk of contracting the disease, concerns about transmitting the infection to family members and colleagues, and a reduced workload, as well as a reduced income, have caused many health professionals to develop various psychological problems, such as anxiety attacks, fear, burnout syndrome, and depression [32, 33]. This reflects the importance of carrying out leisure and rest activities to relieve stress, especially in times of crisis.

To assess whether the perception of negative impacts on personal life varied among participants, a series of cross‐tabulation analyses was conducted against sex, professional experience, and specialty. For impacts with sufficient sample counts, the chi‐square test of independence was used, while Fisher’s exact test was employed for those with low frequencies. Across all analyses, no statistically significant associations were found. The association analysis, supplemented by the TOST for equivalence using a predefined practical relevance margin of ∣Φ∣ < 0.20 or ∣V∣ < 0.20, confirmed the statistical non‐association between the perceived negative impacts and the participants’ characteristics across all variables.

Specifically, the perception of damage to mental health, general health complications, and social isolation did not differ significantly based on sex, professional experience, or specialty. For damage to mental health, while the observed effect size was small (Φ between 0.006 and 0.174), the analysis of the 95% CIs indicated that formal equivalence could not be concluded for the relationship with sex and professional experience. This was because the upper limit of the 95% CI exceeded the 0.20 margin, preventing the definitive rejection of a clinically relevant effect in the population.

The reported experiences of loss of family members, loss of purchasing power, and overload of obligations, although rarely reported, were also found to be statistically homogeneous across all demographic and professional groups. These findings collectively indicate that the negative repercussions of the pandemic on personal life were perceived uniformly throughout the entire sample, with no statistical distinction based on the characteristics of the participants.

By contrast, the following positive aspects were mentioned: an increased sense of community, where people began to care more about others; the acquisition of new knowledge, since Dentistry is one of the professions with the highest risk of coronavirus contamination, and there was a need to seek more information about COVID‐19 and its consequences; the acquisition or improvement of habits—improved cleaning habits in all areas; better personal hygiene habits, such as more attention to hand hygiene, the use of 70% alcohol; greater attention paid to measures that prevented taking contamination home (decontamination of personal objects, care of clothes and shoes used during care); and increased self‐care in environments with large crowds.

It was observed that the pandemic led professionals to reflect more on their lifestyle, practice gratitude, reduce the stressful pace, and reevaluate personal priorities. They began to value life more and spend more time with their families. The COVID‐19 scenario also encouraged greater care for physical and mental health.

Some reported adopting new habits, such as sanitizing their groceries and stores, cleaning their shoes, and taking a shower every time they arrived home, as well as wearing a mask at all times in closed and/or crowded places. Others began avoiding closed and poorly ventilated places and stopped going to places they used to frequent, such as restaurants and parties.

## 4. Conclusions

Due to the political scenario of uncertainty and tension surrounding the measures adopted to control COVID‐19 during the pandemic, healthcare workers have experienced an intense sense of insecurity. The pandemic has significantly affected the lives of these professionals, intensifying stress and fear, promoting a constant state of alert, and causing considerable damage to their mental and physical health.

The new biosafety protocols implemented after the onset of the pandemic have proven to be effective and must be maintained to ensure a safer work environment for both patients and healthcare professionals. In this way, it is possible to reduce stress and insecurity during dental care.

With the advancement of vaccination against COVID‐19 and the resumption of elective dental care in Manaus, it is essential that healthcare managers include planning strategies aimed at improving the mental and general health conditions of SUS professionals. This measure is crucial to minimizing the negative impacts that the pandemic may have caused in the lives of these workers.

The authors recommend that the results from this study, with a limited sample size, should not be generalized beyond the specific group of people studied.

## Conflicts of Interest

The authors declare no conflicts of interest.

## Funding

This research did not receive any specific grant from funding agencies in the public, commercial, or not‐for‐profit sectors.

## Data Availability

The data that support the findings of this study are available on request from the corresponding author. The data are not publicly available due to privacy or ethical restrictions.
